# Suicidality and depression among adult patients admitted in general medical facilities in Kenya

**DOI:** 10.1186/1744-859X-9-7

**Published:** 2010-02-12

**Authors:** David M Ndetei, Lincoln I Khasakhala, Victoria Mutiso, Anne W Mbwayo

**Affiliations:** 1 Africa Mental Health Foundation, Nairobi, Kenya; 2 Department of Psychiatry, University of Nairobi, Nairobi, Kenya

## Abstract

**Aim:**

To document Beck Depression Inventory (BDI) II suicidal symptoms among patients admitted to Kenyan non-psychiatric general medical facilities

**Methods:**

All consenting adults admitted within a period of 4 weeks at 10 general medical facilities in Kenya were interviewed for suicidal symptoms and depression using the BDI-II.

**Results:**

In all, 2,780 patients responded to item 9 (suicidal symptoms of the BDI-II). The prevalence of all BDI-II suicidal symptoms combined was 10.5%. Thoughts of 'killing oneself but have not carried them out' accounted for 9% of the suicidal symptoms. The younger age group had the highest prevalence of suicidal symptoms and the oldest age group had the least prevalence of suicidal symptoms. The more depressed the patients were on the overall BDI-II score, the higher the prevalence of suicidal symptoms.

**Conclusion:**

On average 1 out of 10 of the patients had suicidal symptoms, more so in younger than the older people and in the more depressed. These symptoms had not been clinically recognised and therefore not managed. This calls for clinical practice that routinely enquires for suicidal symptoms in general medical wards.

## Background

Depression is the leading mental disorder associated with suicide [1] especially if there is hopelessness and comorbid acute psychosocial stressors [2]. Physical conditions and depression are often comorbid [3,4]

Undiagnosed depression has been shown to be highly prevalent in Kenyan general medical facilities [4]. Over 25 years ago, Mengech and Dhadphale [5] found a 3.4% attempted suicide rate amongst patients referred from Kenyatta National General Medical facilities to a psychiatric clinic within the same hospital. To date, no study in Kenya has attempted to document the prevalence of suicide symptomatology in patients attending general medical facilities and how these suicide symptoms are associated with depression. This study aims to document Beck Depression Inventory-II (BDI) suicidal symptoms [6] (suicidal thoughts, ideation and plans) among patients admitted to Kenyan non-psychiatric general medical facilities.

## Methods

This was a cross-sectional descriptive study conducted at 10 health facilities selected to represent different operational levels of healthcare provision in Kenya, ranging from the lowest (health centres) to the highest (a national teaching and referral hospital) [4]. All the facilities chosen offer both inpatient and outpatient services, apart from health centres which offer outpatient services only. Psychiatric units in hospitals where mental health services are offered were excluded from the study. Systemic sampling was used to recruit respondents in the facilities, where every third patient either as an outpatient or inpatient was selected. The ethical issues and other exclusion criteria have been described in detail previously [4].

The data was collected by fourth-year and fifth-year medical students trained by the principal investigator, Prof DM Ndetei. The sociodemographic data were extracted from the case notes using a structured format. The 21-item BDI II for adults [6], designed to measure depressive symptoms commensurate with the diagnostic criteria for depressive disorder outlined in the Diagnostic and Statistical Manual of Mental Disorders, fourth edition (DSM-IV), [7] was used to score for depression and suicidal symptoms. The latter were scored as follows: 0 = I don't have any thoughts of killing myself; 1 = I have thoughts of killing myself but have not carried them out; 2 = I would like to kill myself; 3 = I would kill myself if I had the chance.

The cut-off points on the BDI-II for mild to severe depression applied in this study were those cut-off points for patients with physical conditions in medical facilities [8 m 9]. Descriptive statistics were generated using SPSS version 16 (SPSS, Chicago, IL, USA).

## Results

### Response rate

A total of 2,797 respondents were recruited into the study, with full sociodemographic data for 2,780 (99.4%) respondents extracted from the case notes. They were also able to complete question 9 on the BDI-II. In all, 91.5% (n = 2,543) of respondents completed the whole BDI-II.

### Sociodemographic variables and suicide symptoms

The severities of suicidal symptoms according to the sociodemographic variables of the 2,780 patients who responded to item 9 with the 4 possible scores (that is, 0, 1, 2, 3) for suicide symptoms are summarised in Table 1.

**Table 1:** Beck Depression Inventory (BDI)-II suicidal symptoms according to sociodemographic characteristics, n (%)

	BDI-II score				Total	*P *value
			
	0	1	2	3		
Age in years:						
18 to 20	247 (85.5%)	36 (12.5%)	3 (1%)	3 (1%)	289 (10.4%)	NS
	
21 to 25	533 (90.8%)	45 (7.7%)	6 (1.0%)	3 (0.5%)	587 (21.1%)	
	
26 to 30	530 (89.8%)	54 (9.2%)	2 (0.3%)	4 (0.7%)	590 (21.2%)	
	
31 to 45	701 (88.6%)	80 (10.1%)	6 (0.8%)	4 (0.5%)	791 (28.5%)	
	
46 to 60	351 (91.1%)	25 (6.5%)	6 (1.6%)	3 (0.8%)	385 (13.9%)	
	
61 to 75	104 (92.0%)	8 (7.1%)	0 (0%)	1 (0.9%)	113 (4.1%)	
	
>75	23 (92%)	1 (4%)	1 (4%)	0 (0%)	25 (0.9%)	
	
Total	2,489 (89.5%)	249 (9.0%)	24 (0.9%)	18 (0.6%)	2,780 (100%)	

Sex:						

Male	1,148 (89.4%)	116 (9.0%)	12 (1%)	8 (0.6%)	1,284 (46.2%)	NS
	
Female	1,341 (89.6%)	133 (8.9%)	12 (0.8%)	10 (0.7%)	1,496 (53.8%)	
	
Total	2,489 (89.5%)	249 (9.0%)	24 (0.9%)	18 (0.6%)	2,780 (100%)	

Religion:						

Christian	2,293 (89.9%)	217 (8.5%)	22 (0.9%)	15 (0.7%)	2,547 (91.6%)	χ^2 ^= 38.765, df = 12, *P *< 0.000
	
Muslim	87 (82.8%)	15 (14.3%)	1 (1%)	2 (1.9%)	105 (3.8%)	
	
Catholic	89 (92.7%)	6 (6.3%)	0 (0%)	1 (1%)	96 (3.5%)	
	
Hindu	2 (50%)	2 (50%)	0 (0%)	0 (0%)	4 (0.1%)	
	
Others	18 (57.1%)	9 (32.1%)	1 (3.8%)	0 (0%)	28 (1.0%)	
	
Total	2,489 (89.5%)	249 (9%)	24 (0.9%)	18 (0.6%)	2,780 (100%)	

Marital status:						

Single	863 (88.3%)	99 (10.1%)	10 (1%)	5 (0.6%)	977 (35.1%)	χ^2 ^= 48.453, df = 18, *P *< 0.000
	
Married	1,499 (91.0%)	129 (8.8%)	11 (0.7%)	8 (0.5%)	1,647 (59.2%)	
	
Polygamous	33 (84.6%)	5 (12.8%)	0	1 (2.7%)	39 (1.4%)	
	
Cohabiting	14 (77.7%)	3 (16.7%)	1 (5.6%)	0 (0%)	18 (0.6%)	
	
Separated	55 (86.0%)	6 (9.3%)	1 (1.6%)	2 (3.1%)	64 (2.3%)	
	
Divorced	20 (69.0%)	6 (20.7%)	1 (3.4%)	2 (6.9%)	29 (1.0%)	
	
Widowed	5 (83.3%)	1 (16.7%)	0 (0%)	0 (0%)	6 (0.2%)	
	
Total	2,489 (89.5%)	249 (9%)	24 (0.9%)	18 (0.6%)	2,780 (100%)	

Level of education:						

None	83 (94.3%)	5 (5.7%)	0 (0%)	0 (0%)	88 (3.2%)	NS
	
Primary	831 (89.6%)	81 (8.7%)	8 (0.9%)	7 (0.8%)	927 (33.3%)	
	
Secondary	1,025 (88.7%)	112 (9.7%)	9 (0.8%)	9 (0.8%)	1,155 (41.5%)	
	
College	458 (90.3%)	43 (9.5%)	5 (1.3%)	1 (0.2%)	507 (18.2%)	
	
University	92 (89.3%)	8 (7.8%)	2 (1.9%)	1 (1%)	103 (3.7%)	
Total	2,489 (89.5%)	249 (9%)	24 (0.9%)	18 (0.6%)	2,780 (100%)	

BDI-II scores: 0 = I do not have thoughts of killing myself; 1 = I have thoughts of killing myself, but I'd not carry them out; 2 = I'd like to kill myself; 3 = I'd kill myself if I had a chance. Education: primary = 1 to 8 years of formal education; secondary = 9 to 12 years of formal education; college and university = post secondary education.

df = degrees pf freedom; NS = not significant.

The overall prevalence of suicidal symptoms (scores 1, 2 and 3) was 10.5% (n = 291), with the frequency decreasing from scores of 1 to 3.

The highest prevalence of 14.5% suicidal symptoms was in the youngest age group (18 to 20) and least (8.0%) in those over 75 years. The genders were similar. The Catholic religion had the least prevalence, of 7.1%. Divorced marital status had the highest prevalence (20.7%) as opposed to 7.9% in the married group. No education or low levels of education had the least prevalence at 3.7% compared to other levels of education. Having no children and having few children (1 to 2 children) was associated with a higher prevalence of suicide symptoms.

### Total score on BDI-II vs severity of suicide symptoms

A total of 2,543 participants completed all the items of BDI-II including item 9, thus allowing crosstabulation of degree of severity of depression and the different scores for item 9 (suicide symptoms) (Table 2).

**Table 2:** Total Beck Depression Inventory (BDI)-II scores vs severity of suicide symptoms

	BDI-II score				Total of 1,2,3	Total	*P *value^a^
				
	No	Yes					
				
	0	1	2	3			
0 to 13: no depression	1,875 (96.1%)	73 (3.7%)	3 (0.15%)	1 (0.05%)	3.9	1,952 (100%)	χ^2 ^= 6.976E^2a^, df = 9, *P *< 0.000
	
14 to 19: mild depression	270 (81.1%	57 (17.1%)	3 (0.9%)	3 (0.9%)	18.9	333 (100%)	
	
20 to 28: moderate depression	127 (67.9%)	56 (30%)	3 (1.6%)	1 (0.5%)	32.1	187 (100%)	
	
29 to 63: severe depression	23 (32.4%)	29 (40.8%)	10 (14.1%)	9 (12.7%)	67.6	71 (100%)	

Total	2,295 (90.2%)	215 (8.5%)	19 (0.7%)	14 (0.6%)		2,543 (100%)	

BDI-II scores: 0 = I do not have thoughts of killing myself; 1 = I have thoughts of killing myself, but I'd not carry them out; 2 = I'd like to kill myself; 3 = I'd kill myself if I had a chance.

^a^Frequency of suicide symptoms significantly increased with severity of depression.

df = degrees of freedom.

Among the patients who scored for mild depression on the BDI-II (total score of 14 to 19), 18.9% (n = 63) had suicidal symptoms of varying severity; those who scored for moderate depression (total score of 20 to 28), 32.1% had suicidal symptoms of varying degrees, but majority (67.5%) n = 48 who had severe depression (score of >28) had suicidal symptoms whereas of those who had normal scores (total score of 0 to 13) on the BDI-II only 3.9% (n = 77) had suicidal symptoms.

## Discussion

The findings of this study must be considered in light of various caveats. First, this was a cross-sectional study in patients being managed for medical conditions. The nature of the medical conditions and the severity of those conditions could have had an effect on the severity of either or both the depression and the suicidal symptoms. It is therefore conceivable that the severity of the depression and the symptoms of suicide would have varied if these were pegged to specific medical conditions and on varying severity of those specific physical conditions. Further, the severity of the depression and the suicidal symptoms could vary with duration of a given specific condition. The findings of this study can therefore at best be regarded as a cumulative average dependent on multiple factors.

In mitigation however, this study was primarily focused on the prevalence of suicidal symptoms and not association with specific underlying diagnosis, whether psychiatric and/or physical diagnoses or neither. Further, depression is the commonest cause of suicide symptoms and given that these patients were being managed by non-psychiatrists, the BDI-II was chosen as the screener for depression because it has been found to be useful in general non-psychiatric facilities [8,9] and can be self-administered or administered by non-psychiatrist.

A further limitation of this study is the fact that item 9 of the BDI-III contributes to the overall scoring for BDI-II, although it was selected to gauge how it is associated with overall depression. Another limitation is that the psychometric properties of the BDI-II in the Kenyan sociocultural context, and more specifically in general medical settings, have not been described, but this is mitigated by the fact that the BDI-II has been used extensively in similar settings and producing results similar to those found in other parts of the world studying similar psychiatric populations [4]. When suicidal ideation is taken alone, the 9.0% prevalence is similar to the 9.1% prevalence in general medical facilities in South Africa [10].

On a positive note, the response rate for all variables was high, suggesting a high interest in patients to participate. This was despite the fact that voluntary and informed consent was obtained from all those who were well enough [4], and were therefore under no obligation to participate in the study.

With all the above caveats in mind the results can be discussed.

The findings of this study are noteworthy in that they demonstrate more similarities than dissimilarities with findings across the globe.

The 10.5% overall prevalence of suicidal symptoms in this population of general medical patients in Kenya compares favourably with the 11.6% found in an emergency treatment centre in Texas, USA [11]. However, as will be discussed below, this 10.5% included suicidal symptoms in both depressed and non-depressed patients although the depressed patients had most of the symptoms. That there were no gender differences is similar to the findings from two African countries (Ghana and Uganda) and one European country (Norway) [12] but in contradiction to most studies that have found a higher prevalence in females [10,13-16] including studies in the neighbouring Uganda [17] and Ghana [18].

The sociodemographic risk factors associated with depression are similar to those found by Nock *et al*. across 17 countries using the World Health Organization World Mental Health Survey Initiative [13]. These are young age and unmarried status (single, separated, divorced or widowed). That suicidal symptoms were associated with divorced marital status was also found by Kposwa [19]. However, unlike the finding of Nock *et al*. that few years of education was a risk factor, this study found that no education and low level of education seemed to be protective against suicidal symptoms compared with higher level of education. It may be that a higher level of education raises expectation of career opportunities, which could result in depression if not fulfilled in an environment of high unemployment.

The finding that there were differences in religion, with Christianity having the least prevalence is different from that of Eshun [18], who found no differences in both Ghana and America. However, these Kenyan findings could be an artefact of the small numbers of other religions, most scoring for suicidal symptoms and possibly only severe symptoms would find expression because of strong taboos against suicide in the respective religions.

The finding that 63.9% of those with moderate depression were suicidal is indeed a reflection of the untreated depression in 42.0% of those with depression in the facilities studied [4], which is also in agreement with Schlebusch [20] that untreated depression is one of the major causes of suicide.

In conclusion, and despite the limitations of this study, these Kenyan results do not have any findings different from what is already known from the common global pool of data. However, they do add a voice to the global similarities in mental disorders and depression in particular, despite the global inequities in resources to address mental health disorders. For Kenya in particular and other socioeconomically similar countries in Africa, those findings clearly demonstrate the need for appropriate practices and policies to increase awareness of, and screen for, depression and suicide symptoms routinely in clinical practice and to look for innovative interventions given the highly limited resources [21].

## Competing interests

The authors declare that they have no competing interests.

## Authors' contributions

DMN contributed to the conception and design of the study and was involved in drafting the manuscript and revising it critically for intellectual content, and also in the training of the data collectors. LIK participated in acquisition, analysis and interpretation of data and was involved in drafting the manuscript and revising it critically for intellectual content. VNM participated in acquisition, analysis and interpretation of data and was involved in drafting the manuscript. AWM participated in drafting and editing the manuscript.
